# Limb salvage versus amputation in patients with osteosarcoma of the extremities: an update in the modern era using the National Cancer Database

**DOI:** 10.1186/s12885-020-07502-z

**Published:** 2020-10-14

**Authors:** Daniel R. Evans, Alexander L. Lazarides, Julia D. Visgauss, Jason A. Somarelli, Dan G. Blazer, Brian E. Brigman, William C. Eward

**Affiliations:** 1 Duke University School of Medicine, Durham, NC USA; 2 Department of Orthopaedic Surgery, Duke University Hospital, 311 Trent Drive, Durham, NC 27710 USA; 3 Duke Cancer Institute, Duke University Hospital, Durham, NC USA

**Keywords:** Limb salvage surgery, Amputation, Osteosarcoma, Osteosarcoma of the extremities, Overall survival

## Abstract

**Background:**

Historically, amputation was the primary surgical treatment for osteosarcoma of the extremities; however, with advancements in surgical techniques and chemotherapies limb salvage has replaced amputation as the dominant treatment paradigm. This study assessed the type of surgical resection chosen for osteosarcoma patients in the twenty-first century.

**Methods:**

Utilizing the largest registry of primary osteosarcoma, the National Cancer Database (NCDB), we retrospectively analyzed patients with high grade osteosarcoma of the extremities from 2004 through 2015. Differences between patients undergoing amputation and patients undergoing limb salvage are described. Unadjusted five-year overall survival between patients who received limb salvage and amputation was assessed utilizing Kaplan Meier curves. A multivariate Cox proportional hazard model and propensity matched analysis was used to determine the variables independently correlated with survival.

**Results:**

From a total of 2442 patients, 1855 underwent limb salvage and 587 underwent amputation. Patients undergoing amputation were more likely to be older, male, uninsured, and live in zip codes associated with lower income. Patients undergoing amputation were also more likely to have larger tumors, more comorbid conditions, and metastatic disease at presentation. After controlling for confounders, limb salvage was associated with a significant survival benefit over amputation (HR: 0.70; *p* < 0.001). Although this may well reflect underlying biases impacting choice of treatment, this survival benefit remained significant after propensity matched analysis of all significantly different independent variables (HR: 0.71; *p* < 0.01).

**Conclusion:**

Among patients in the NCDB, amputation for osteosarcoma is associated with advanced age, advanced stage, larger tumors, greater comorbidities, and lower income. Limb salvage is associated with a significant survival benefit, even when controlling for significant confounding variables and differences between cohorts.

## Background

Osteosarcoma, an aggressive bone cancer, is the most common primary malignancy of bone [1–4]. A cancer that was previously considered a death sentence, improvements in chemotherapy regimens and surgical treatment have dramatically improved 5 year overall survival for non-metastatic osteosarcoma from 22% in 1950 to 70% presently [4–6]. The previous gold standard for surgical treatment was amputation; however, with advances in surgical procedures, limb salvage replaced amputation as the dominant treatment paradigm [5, 6]. In 1984, the National Institute of Health deemed limb salvage an equal treatment option to amputation, leading to some debate the future role for amputation [6–8].

Efforts to establish the benefits of limb salvage over amputation have yielded conflicting results in relation to survival, functional recovery, and psychological effects [6, 7]. Due to the rarity of osteosarcoma, many of the studies suffer in quality because they have had small sample sizes, assessed different outcome variables, and have had limited scopes, all of which have restricted our ability to perform effective meta-analyses. Existing meta-analyses [6, 9] that have been performed have found that limb salvage improved survival; however, all of these analyses remain limited by low study numbers and sample sizes, and there is a continued need for studies that leverage larger data sets. With regard to making associations between type of surgery and survival, there is significant bias at play. Those patients who receive amputations often have larger tumors, other patient factors which make limb salvage inappropriate, or mitigating disease features (such as pathologic fracture or superimposed infection) which may lead to poorer outcomes.

The National Cancer Database (NCDB) is a clinical oncology database that captures 70% of all new cancer diagnoses in the United States [10]. While osteosarcoma is rare, this database provides an opportunity to investigate this cancer with adequate sample size while better controlling for confounders by normalizing variables and outcomes across cohorts [10]. In the present study we used the National Cancer Database to ask the questions: “Who receives an amputation in recent decades?” and “How does the amputation population differ from the limb salvage population?” We also sought to determine if there is a difference in survival in patients that receive limb salvage surgery versus those that receive amputation for osteosarcoma when we attempt to control for confounding variables.

## Methods

### Study cohort

**Fig. 1:**
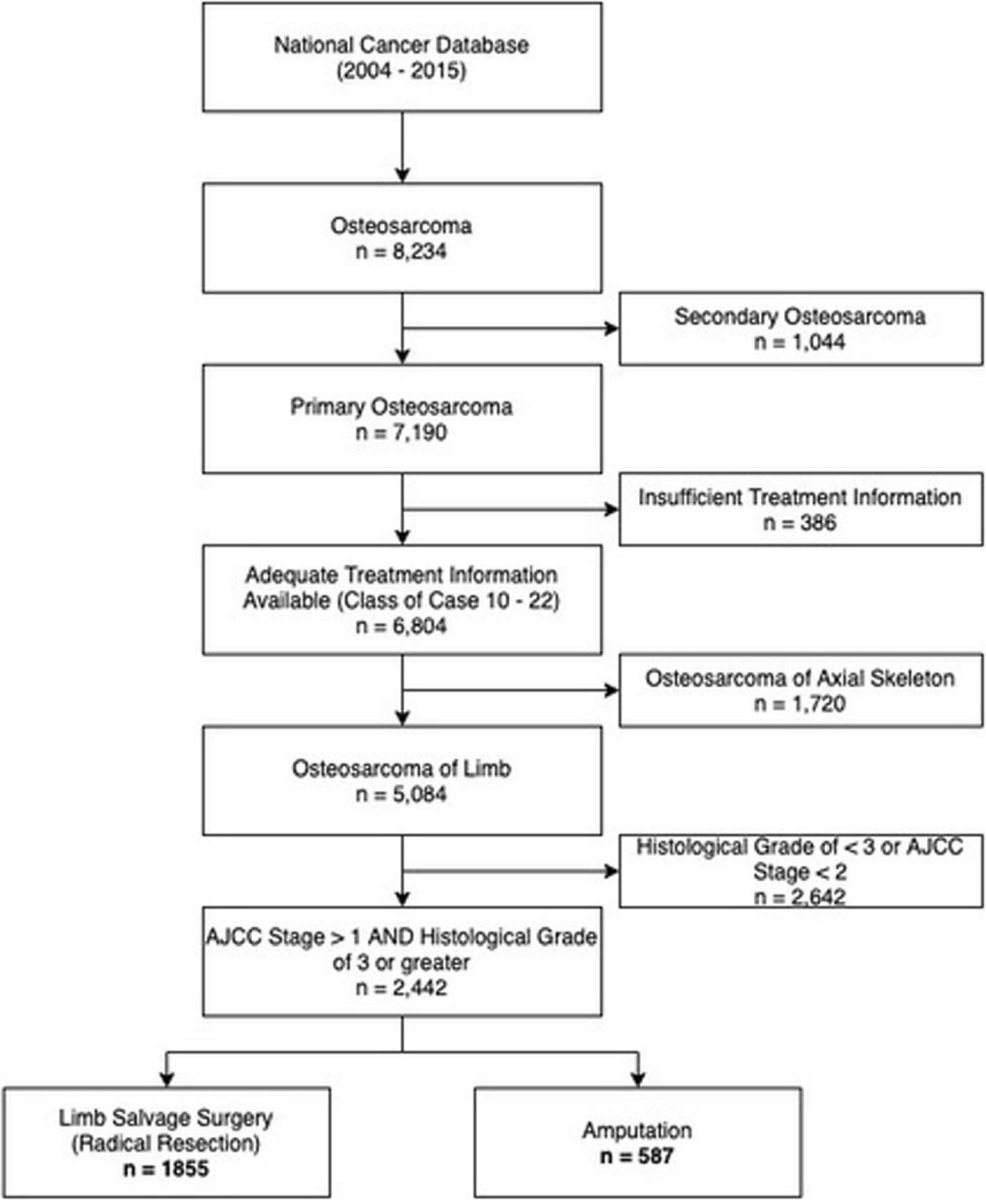
Cohort selection flow chart

We retrospectively queried the NCDB for all osteosarcoma cases between 2004 and 2015. Cases met inclusion criteria if they had the following features: (1) undergone surgical resection or amputation for osteosarcoma; 2) osteosarcoma was the primary cancer; 3) the primary tumor site was in the extremities; 4) and had histological diagnosis with a histological grade of 3 or greater (Fig. 1). Cases were excluded that had incomplete or missing treatment data. Patients were also excluded that did not undergo radical resection or amputation.

### Variables

The demographic factors we considered included age, sex, race, insurance status, Zip code income quartile, Zip code educational quartile, rural/urban, distance from hospital in miles, and year of diagnosis. Age was considered as both a continuous and categorical variable, which was split into three categories (< 18, 18–40, > 40). Race was classified as Caucasian, African-American, Latinx/Hispanic, Asian/Pacific Islander, and other. Insurance status was classified as uninsured, insured, Medicaid, and Medicare. Year of diagnosis was parsed into before 2006, 2006–2010, and 2011–2015. Clinicopathological factors included American Joint Commission on Cancer (AJCC) clinical stage at diagnosis, upper/lower extremity, long/short bone, tumor size (cm), metastases at diagnosis, comorbidities at diagnosis, adjuvant chemotherapy, chemotherapy/surgery treatment sequence, surgical margins, unplanned readmission after surgery, length of inpatient stay after surgery, days from diagnosis to surgery, and number of days from diagnosis to treatment commencing. The primary outcome of interest was overall survival (OS).

### Statistical analysis

Utilizing site-specific surgical codes, patients were separated into the following two cohorts: 1) those who received radical resection with limb salvage and 2) those who received amputation. The demographic and clinicopathologic characteristics of cohorts were compared using a student’s T-test for continuous variables and Chi^2^ for categorical variables. Unadjusted overall survival (OS) was estimated using the Kaplan Meier method, with statistical comparisons based on the log-rank test. Univariate analyses to test for statistically robust associations between OS and patient demographic, clinical, and treatment were assessed via Kaplan Meier; using the log rank test if categorical or a univariate Cox regression if continuous. A multivariate Cox proportional hazard model was then used to determine OS adjusted to control for significant differences between study groups and potential confounding. Demographic and clinicopathological variables of age, sex, insurance status, upper/lower limb location, AJCC stage, tumor size, metastases at diagnosis, comorbidities, surgical margins, and adjuvant chemotherapy were potential confounders and were controlled for in the multivariate Cox analysis and were used to create the matched propensity score analysis. To account for clinician selection bias between cohorts a logit matched propensity score analysis was used to match the two cohorts on all demographic and clinicopathological variables that were significantly different, with a caliper distance of less than 0.1 standard deviations. A Cox regression was then used to analyze the hazard ratio of the matched cohorts.

### Ethical approval

Ethical approval was granted by the Duke IRB Pro00045337. Statistical analyses were conducted using STATA 15 (StataCorp LLC; College Station, TX) and statistical significance was determined at a *p*-value < 0.05.

## Results

### Cohort characteristics

A total of 2442 patients met the inclusion criteria. The patient cohort had a median age of 18 (IQR 13–32), and a small majority (60.3%) of the patients were male. Average tumor size was 11.9 cm (11.5–12.4 cm) with metastases present in 405 (16.8%) of the patients. Of the patients that met the inclusion criteria, 1885 patients underwent a radical, limb-sparing resection while 587 patients underwent an amputation.

**Fig. 2:**
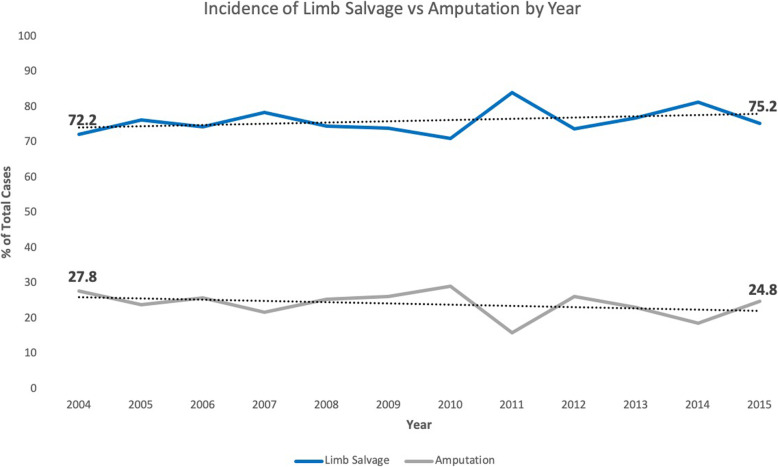
Incidence of limb salvage vs amputation over time

There were significant demographic and clinicopathological differences between the two cohorts. Demographically, patients that underwent amputation were significantly more likely to be older, male, uninsured, and live in lower income Zip codes (*p*</=0.03) (Table 1). Clinicopathologically, patients that underwent amputation were significantly more likely to have an earlier year of diagnosis, higher stage at presentation, larger tumors, metastatic disease at presentation, more baseline comorbidities at diagnosis, not receive adjuvant chemotherapy, and have negative surgical margins (*p*</=0.023) (Table 2). The incidence of limb salvage vs amputation increased over the study period (Fig. 2).

**Table 1:** Demographic characteristics of patients undergoing limb salvage surgery vs. amputation

	Type of Surgery						***p*** value
	Limb Salvage Surgery		Amputation		Total		
	n	%	n	%	n	%	
**Age**; median (IQR)	17	(13–28)	21	(14–45)	18	(13–32)	**< 0.0001**
< 20	1135	61.2%	267	45.5%	1402	57.4%	**< 0.0001**
20–39	399	21.5%	152	25.9%	551	22.6%	
40–59	199	10.7%	98	16.7%	297	12.2%	
60+	122	6.6%	70	11.9%	192	7.9%	
**Sex**							
Male	1095	59.0%	377	64.2%	1472	60.3%	**0.025**
Female	760	41.0%	210	35.8%	970	39.7%	
**Race**							
White	1398	75.4%	436	74.3%	1834	75.1%	0.923
Black	295	15.9%	98	16.7%	393	16.1%	
Asian/Pacific Islander	80	4.3%	28	4.8%	108	4.4%	
Other/Unknown	82	4.4%	25	4.3%	107	4.4%	
**Insurance Status**							
Uninsured	69	3.9%	33	5.7%	102	4.3%	**< 0.0001**
Insured	1207	67.5%	316	54.9%	1523	64.4%	
Medicaid	418	23.4%	168	29.2%	586	24.8%	
Medicare	94	5.3%	59	10.2%	153	6.5%	
**Zipcode Income**							
< $38,000	345	18.8%	105	18.2%	450	18.7%	**0.002**
$38,000–$47,999	398	21.7%	158	27.4%	556	23.1%	
$48,000–$62,999	476	26.0%	163	28.3%	639	26.5%	
$63,000+	615	33.5%	150	26.0%	765	31.7%	
**Zipcode Education**							
21% or more	381	20.8%	112	19.4%	2009	85.1%	0.258
13–20.9%	443	24.1%	163	28.3%	316	13.4%	
7–12.9%	568	31.0%	169	29.3%	36	1.5%	
< 7%	443	24.1%	132	22.9%	575	23.8%	
**Urban**							
Metro	1539	85.8%	470	82.7%	2817	85.2%	0.052
Urban	232	12.9%	84	14.8%	436	13.2%	
Rural	22	1.2%	14	2.5%	54	1.6%	
**Distance from Hospital (Miles);** mean (95% CI)	70.4	(63.30–77.53)	59.4	(51.20–67.65)	67.8	(62.03–73.55)	0.1103
**Year of Diagnosis**							
< 2006	279	15.0%	97	16.5%	376	15.4%	0.067
2006–2010	748	40.3%	260	44.3%	1008	41.3%	
2011–2015	828	44.6%	230	39.2%	1058	43.3%	
**Total**	1855	75.96%	587	24.04%	2442	100%

**Table 2:** Clinicopathological variables of patients undergoing limb salvage surgery vs. amputation

	Type of Surgery						***p*** value
	Limb Salvage Surgery		Amputation		Total		
	n	%	n	%	n	%	
**Location**							
Lower Limb	1536	82.8%	506	86.2%	2042	83.6%	0.053
Upper Limb	319	17.2%	81	13.8%	400	16.4%	
**Long vs Short Bone**							
Short Bone	51	2.8%	49	8.5%	100	4.1%	***p*** **< 0.001**
Long Bone	1789	97.2%	527	91.5%	2316	95.9%	
**AJCC Clinical Stage**							
Stage 2A or 2B	1509	81.3%	438	74.6%	1947	79.7%	***p*** **= 0.002**
Stage 3	63	3.4%	25	4.3%	88	3.6%	
Stage 4A or 4B	283	15.3%	124	21.1%	407	16.7%	
**Osteosarcoma Type**							
Osteosarcoma NOS	1216	66.1%	407	70.7%	1623	67.2%	0.059
Chondro/Fibroblastic	370	20.1%	111	19.3%	481	19.9%	
Telangiectatic	78	4.2%	24	4.2%	102	4.2%	
Central	108	5.9%	24	4.2%	132	5.5%	
Surface/Juxtacortical	68	3.7%	10	1.7%	78	3.2%	
**Tumor Size (cm);** mean (95% CI)	11.6	11.0–12.1	13.1	12.2–13.9	11.9	11.5–12.4	**0.005**
< 8 cm	547	30.6%	132	23.0%	679	28.7%	***p*** **< 0.001**
> 8 cm	1242	69.4%	442	77.0%	1684	71.3%	
**Presence of Metastases at Dx**							
No metastases	1549	84.7%	454	78.3%	2003	83.2%	***p*** **< 0.001**
metastases	279	15.3%	126	21.7%	405	16.8%	
**Comorbidities at Dx**							
No comorbidities	1714	92.4%	520	88.6%	2234	91.5%	**0.004**
1 or more	141	7.6%	67	11.4%	208	8.5%	
**Received Adjuvant Chemotherapy**							
No	81	4.4%	62	10.6%	143	5.9%	***p*** **< 0.001**
Yes	1762	95.6%	521	89.4%	2283	94.1%	
**Sequence of Adjuvant Treatment**							
Before	558	37.3%	170	38.7%	728	37.6%	***p*** **< 0.001**
After	162	10.8%	88	20.0%	250	12.9%	
Before+After	777	51.9%	181	41.2%	958	49.5%	
**Surgical Margins**							
Clear	1617	93.7%	541	96.6%	2158	94.4%	***p*** **= 0.010**
Positive	108	6.3%	19	3.4%	127	5.6%	
**Unplanned Readmission After Surgery**							
No Unplanned Readmiss	1727	96.2%	548	95.6%	2275	96.1%	0.537
Unplanned Readmission	68	3.8%	25	4.4%	93	3.9%	
**Inpatient Days after Surgery;** mean (95% CI)	6.7	6.3–7.1	6.3	5.6–7.1	6.6	6.3–7.1	0.3706
**Days from Dx to Surgery;** mean (95% CI)	96.1	94.1–98.1	91.8	87.6–96.1	95.1	93.3–96.9	**0.049**
**Days from Dx to Treatment Commencing;** mean (95% CI)	20.5	19.4–21.5	22.4	20.3–24.5	20.9	20.0–21.9	0.0796
**Total**	1855	75.96%	587	24.04%	2442	100%

### Univariate survival analysis

Unadjusted univariate Kaplan Meier and Cox analyses found a significant difference in OS between the two cohorts (log rank *p* < 0.001). The five-year overall survival rates were 67.8 and 53.7% for limb salvage and amputation, respectively. The unadjusted hazard ratio for amputation was 1.66 (*p* < 0.001).

On univariate analysis improved survival after limb salvage was significantly associated with patients that were younger, female, insured, with lower AJCC stage, smaller tumor size, no metastases, received chemotherapy, had negative surgical margins and a shorter time from diagnosis to treatment commencing (*p*</=0.004). Similarly, improved survival after amputation was significantly associated with patients who were younger, from a higher income zip code, insured, had a lower AJCC stage, smaller tumor size, no metastases, received chemotherapy, and a shorter time from diagnosis to surgical treatment (*p*</=0.03). This suggests that the factors associated with improved survival are largely independent from the type of surgery performed.

### Propensity score matched survival analysis

**Fig. 3:**
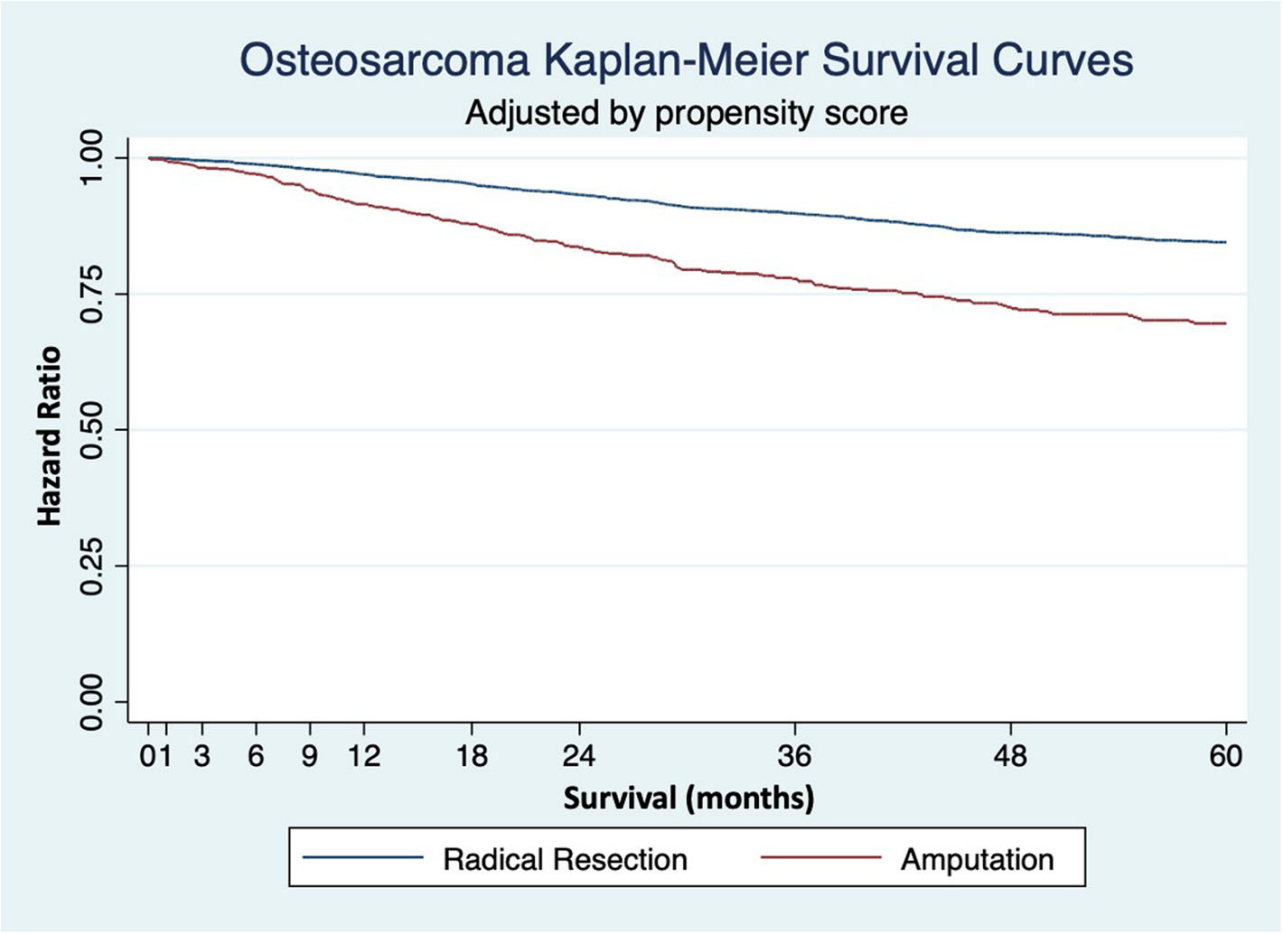
Kaplan Meier adjusted for propensity analysis

Propensity score matching is a multivariate method that estimates the treatment effect size while controlling for the likelihood of receiving a treatment. A matched propensity score analysis was performed as an attempt to control for the likelihood of patients to receive certain treatments based on their specific demographics and tumor characteristics. The matched propensity score analysis demonstrated improved survival with limb salvage, having a five-year overall survival treatment benefit of 10.7% (5.4–16.0%; *p* < 0.001) (Fig. 3). Cox regressions adjusted by linear matched propensity score also revealed similar results with a hazard ratio of 1.4 (*p* < 0.001) for amputation. Within the propensity score matched cohort improved survival was significantly associated with patients that were younger, insurance status, lower AJCC stage, had osteosarcoma of the short bones or the lower limb, smaller tumor size, no metastases, negative surgical margins and received adjuvant chemotherapy (*p*</=0.006) (Table 3).

**Table 3:** Hazard ratios for survival adjusted for by propensity analysis

	Total			Limb Salvage (***n*** = 1316)			Amputation (***n*** = 409)		
	HR	***P***	95% CI	HR	***P***	95% CI	HR	***P***	95% CI
**Surgery Type**				0.71	**0.000**	0.59–0.85	1.42	**0.001**	1.13–1.63
**Age**; median (IQR)	1.02	**0.000**	1.01–1.02	1.02	**0.000**	1.01–1.02	1.01	**0.001**	1.01–1.02
< 20	Reference								
20–39	1.15	0.195	0.93–1.42	1.11	0.413	0.86–1.44	1.12	0.549	0.77–1.64
40–59	1.29	0.056	0.99–1.67	1.33	0.076	0.97–1.82	1.20	0.446	0.75–1.94
60+	2.33	**0.000**	1.72–3.16	2.57	**0.000**	1.74–3.8	1.94	**0.010**	1.17–3.21
**Sex**									
Male									
Female	0.87	0.111	0.73–1.03	0.79	**0.030**	0.64–0.98	1.10	0.530	0.82–1.49
**Race**									
White	Reference								
Black	0.98	0.834	0.78–1.22	0.82	0.172	0.61–1.09	1.29	0.160	0.9–1.85
Asian/Pacific Islander	1.01	0.974	0.66–1.53	1.14	0.619	0.69–1.88	0.75	0.465	0.35–1.61
Other/Unknown	1.36	0.107	0.94–1.99	1.27	0.321	0.8–2.01	1.63	0.139	0.85–3.1
**Insurance Status**									
Uninsured	0.87	0.452	0.59–1.26	0.87	0.556	0.55–1.39	0.94	0.837	0.5–1.75
Insured	Reference								
Medicaid	0.69	**0.001**	0.55–0.85	0.61	**0.000**	0.47–0.8	0.85	0.38	0.59–1.22
Medicare	1.53	**0.006**	1.13–2.07	1.17	0.461	0.77–1.77	2.29	**0.001**	1.44–3.65
**Zipcode Income**									
< $38,000	Reference								
$38,000–$47,999	0.81	0.099	0.64–1.04	0.65	**0.007**	0.48–0.89	1.17	0.450	0.78–1.76
$48,000–$62,999	0.77	**0.032**	0.6–0.98	0.87	0.342	0.65–1.16	0.62	**0.033**	0.4–0.96
$63,000+	0.92	0.468	0.72–1.16	1.01	0.950	0.76–1.34	0.77	0.250	0.49–1.2
**Zipcode Education**									
21% or more	Reference								
13–20.9%	0.96	0.713	0.75–1.22	0.91	0.551	0.68–1.23	1.06	0.769	0.71–1.6
7–12.9%	0.90	0.356	0.71–1.13	0.94	0.678	0.71–1.25	0.84	0.424	0.56–1.28
< 7%	0.98	0.851	0.76–1.25	1.11	0.484	0.82–1.5	0.73	0.176	0.46–1.15
**Urban**									
Metro	0.49	**0.016**	0.28–0.87	0.48	0.108	0.2–1.17	0.60	0.187	0.28–1.28
Urban	0.52	**0.035**	0.29–0.96	0.55	0.196	0.22–1.37	0.57	0.193	0.25–1.32
Rural	Reference								
**Distance from Hospital (Miles);** mean (95% CI)	1.00	0.547	1–1	1.00	0.536	1–1	1.00	0.982	1–1
**Year of Diagnosis**	1.00	0.771	0.98–1.03	0.99	0.585	0.96–1.03	1.04	0.083	0.99–1.1
< 2006	Reference								
2006–2010	1.06	0.614	0.84–1.33	1.01	0.935	0.77–1.34	1.18	0.414	0.79–1.77
2011–2015	1.01	0.949	0.78–1.3	0.91	0.529	0.67–1.23	1.40	0.138	0.9–2.18
**Location**									
Lower Limb	Reference								
Upper Limb	1.52	**0.000**	1.23–1.89	1.38	**0.013**	1.07–1.78	2.24	**0.000**	1.51–3.33
**Long vs Short Bone**									
Short Bone	Reference								
Long Bone	3.89	**0.000**	2.44–6.19	2.25	**0.007**	1.24–4.06	6.68	**0.000**	3.2–13.95
**AJCC Clinical Stage**									
Stage 1A or 1B									
Stage 2A or 2B	Reference								
Stage 3	1.34	0.195	0.86–2.07	1.70	**0.045**	1.01–2.87	0.79	0.573	0.35–1.79
Stage 4A or 4B	2.82	**0.000**	2.33–3.4	2.88	**0.000**	2.28–3.64	2.73	**0.000**	1.96–3.78
**Osteosarcoma Type**									
Osteosarcoma NOS									
Chondroblastic	0.90	0.324	0.74–1.11	1.07	0.573	0.84–1.37	0.65	**0.023**	0.45–0.94
Fibroblastic	0.63	0.067	0.39–1.03	0.46	**0.024**	0.24–0.9	1.15	0.708	0.56–2.34
Telangiectatic	0.95	0.775	0.65–1.38	0.96	0.870	0.61–1.51	0.99	0.974	0.48–2.03
Central	0.71	0.288	0.38–1.34	0.76	0.461	0.37–1.56	0.79	0.740	0.19–3.2
Surface/Juxtacortical									
**Tumor Size (cm);** mean (95% CI)	1.01	**0.002**	1–1.01	1.00	0.174	1–1.01	1.02	**0.000**	1.01–1.03
< 8 cm	Reference								
> 8 cm	1.68	**0.000**	1.38–2.04	1.44	**0.002**	1.15–1.81	2.27	**0.000**	1.53–3.39
**Presence of Metastases at Dx**									
No metastases	Reference								
metastases	2.93	**0.000**	2.44–3.53	3.00	**0.000**	2.39–3.76	2.79	**0.000**	2.02–3.86
**Comorbidities at Dx**									
No comorbidities	Reference								
1 or more	1.02	0.916	0.77–1.34	0.86	0.429	0.6–1.24	1.30	0.247	0.83–2.01
**Received Adjuvant Chemotherapy**									
No	Reference								
Yes	0.58	**0.001**	0.42–0.79	0.80	0.326	0.51–1.25	0.38	**0.000**	0.24–0.59
**Surgical Margins**									
Clear	Reference								
Positive	2.86	**0.000**	2.11–3.88	3.36	**0.000**	2.41–4.69	1.85	0.116	0.86–3.99
**Unplanned Readmission After Surgery**									
No Unplanned Readmiss	Reference								
Unplanned Readmission	0.69	0.121	0.43–1.1	0.69	0.192	0.4–1.2	0.67	0.374	0.27–1.62
**Inpatient Days after Surgery;** mean (95% CI)	1.00	0.801	0.99–1.01	1.00	0.942	0.99–1.01	1.01	0.164	0.99–1.03
**Days from Dx to Surgery;** mean (95% CI)	1.00	0.602	1–1	1.00	0.144	1–1	1.00	0.321	1–1
**Days from Dx to Treatment Commencing;** mean (95% CI)	1.00	**0.020**	1–1.01	1.01	**0.000**	1–1.01	1.00	0.528	0.99–1

## Discussion

The last half century has given way to amputation for osteosarcoma being replaced with limb salvage surgery. However, there exists little data on the current state of amputation vs. limb salvage in the modern era within large, multi-institutional databases. Our study provides a modern update to the question of “Who receives an amputation?” among patients with high grade osteosarcoma. We found that there were significant differences between those patients who underwent amputation and those patients who underwent limb salvage surgery. Patients who received an amputation were more likely to have a variety of demographic (e.g. lower income, uninsured) and clinicopathologic (e.g. larger tumors, advanced stage) features which are independently associated with poor outcomes. It was therefore not surprising to learn that patients undergoing amputation had poorer overall survival. However, we noted that this difference in survival persisted even when matched propensity score analysis was used to attempt to control for selection bias.

Historically, limb salvage surgery (LSS) with chemotherapy was viewed as an equivalent surgical option to amputation with regard to overall survival. A seminal study by Rougraff et al. investigated 227 patients with nonmetastatic high grade osteosarcoma in a multicenter retrospective review [11]. They found that, compared with amputation, limb salvage resulted in higher rates of reoperation and a higher functional outcome without affecting long-term survival. These findings were supported by a study from Bacci et al. investigating 560 patients with osteosarcoma of the extremities and found no difference in survival between patients treated with limb salvage vs. amputation, finding instead that response to chemotherapy and surgical margins were a much better predictor of local recurrence and overall survival [12]. They concluded that limb salvage is safe at institutions where patients will undergo margin negative surgery with appropriate adjuvant therapies.

A recent study that was published by Traven et al. [13] indicated that, in a propensity matched analysis, amputation was associated with significantly worse survival compared to limb salvage surgery, with a hazard ratio of 1.7 for amputation. Their study similarly utilized advanced statistics to help control for factors that are possible confounders, specifically the propensity to receive certain treatments based on tumor and patient characteristics. Additionally, the SEER database allows for investigation of disease specific survival, an important consideration that is not available in the NCDB. However, there are a number of limitations with this study and many questions remain unanswered. The SEER database only captures 30% of new cancer diagnoses, a factor that raises the concern that this study does not represent the population as a whole. The final study cohort of limb salvage and amputation patients included only 2820 patients. In contrast, the NCDB captures over 70% of new cancer diagnoses annually with strict follow-up requirements. Information about treatment specifics, an important consideration for patients with osteosarcoma, is limited in both the SEER database and the NCDB. Finally, and perhaps most importantly, the SEER database includes data ranging from 1975 to 2016; nearly 60% were inadequately staged and had a grade of “unknown.” Without proper exclusion, this introduces a significant confounding variable, as it stands to reason that the state of cancer care, supportive measures and technical skill has improved between 1975 and 2016. Taken together, these limitations indicate additional studies are required to understand the modern impact of limb salvage surgery versus amputation on survival.

In addition to describing the different patient features associated with amputation versus limb salvage, our study also confirms the importance of chemotherapy, which was found to be a protective factor in a multivariate analysis. Conversely, positive margin status was found to be a negative predictor of outcome in a multivariate analysis. The importance of good chemotherapeutic response and margin-negative surgery in the treatment of high-grade osteosarcoma has been well established in the literature [12, 14, 15] and so these findings were not surprising. We also found that low tumor grade and lower AJCC staging was also prognostic for improved overall survival. These factors have similarly been previously demonstrated as important prognostic factors for survival in osteosarcoma [16, 17]. Taken as a whole, these findings affirm the importance of these factors in the management and treatment of high-grade osteosarcoma.

The NCDB is not without its own limitations. It is not possible to determine whether or not a patient experienced local recurrence of disease – the factor that would be of high interest in concern when comparing limb salvage and amputation for high grade osteosarcoma. It was interesting to note that although limb salvage was found to be equivalent to amputation in the 1980s, there is a clear trend in the NCDB for more amputations to have been performed closer to the year 2004 (the first year of this study) than to year 2015 (the last year of this study). Similar to Traven et al. [13], the present study demonstrated that patients with high grade osteosarcoma who underwent amputation were approximately 1.4 times more likely to experience mortality than patients who underwent limb salvage even in our propensity matched analysis. While it is notable that both our study and the Traven et al. [13] study found a survival benefit associated with limb salvage, and both studies attempted to control for bias, it is important to note that these findings do not determine a causative association. The biases at play – sicker patients, larger tumors, greater comorbidities, and fewer resources (income, health insurance) – are powerful forces and it may be impossible to control for them completely. This latter question, “Does type of surgery impact survival?” is difficult to answer with any database. Many specific factors - poor response to chemotherapy, excessive tumor burden, proximity to neurovascular bundles, local recurrence and generally worse tumor biology – are not captured by the NCDB. Any one of these factors could represent a linkage between type of surgery chosen and disease severity. It is also possible that limb salvage surgery provides, in and of itself, an impact on survival. One might speculate that better functional status, exercise tolerance, self image, and the benefits to physiology associated with these factors may play a role but it is impossible to investigate such features within the NCDB (or any large database).

While this study provides important support as to the modern state of limb salvage surgery versus amputation, it is not without its own limitations. Importantly, this is a retrospective database review. The quality of the database is only as strong as the quality of the data entry. It should be noted that the NCDB employs advanced quality screening metrics and mandates that contributing centers maintain at least 70% follow-up. Nonetheless, it is possible for data to be entered inaccurately. Additionally, the retrospective nature of this database cannot imply causation and is inherently subject to selection bias and possible confounding biases not otherwise addressed in this study. This is notable limitation as there are limits to the “granularity” of the data available in the NCDB. It stands to reason that there are tumor and patient specifics that contribute to the decision for amputation vs. LSS that simply cannot be captured by the basic socioeconomic and tumor/ treatment variables noted in this database. For instance, it stands to reason that patients with extensive neurovascular involvement, poor chemotherapeutic response or for whom life expectancy is not expected to be very long may be precluded from receiving LSS. Despite this, we made every limitation to control for the variables available and provide the most robust data analysis possible using propensity matched methods. Finally, the NCDB lacks specifics regarding resections, such as neurovascular involvement or response to chemotherapy; these may be important considerations in the decision-making towards limb salvage. Nonetheless, we feel that this study is an important update as to the current state of surgical intervention for osteosarcoma; multivariate and propensity score matched statistics were used help control for the retrospective nature of the study and potentially confounding variables to better identify the true impact of limb salvage on overall survival vs. amputation.

Taken together, the strengths and weaknesses of a large database study such as this leads to the question of how to contextualize this information into modern clinical practice. We suggest that, at the very minimum, this study reinforces that limb salvage surgery is at least equivalent to amputation. We also suggest that, when possible, limb salvage should remain the standard of care for patients undergoing management for osteosarcoma of the extremities. Amputation should continue to be chosen if patient and clinical factors make a favorable outcome with LSS unlikely; the mainstay of all conversations around the surgical management of osteosarcoma should involve a patient centered approach discussing the risks and benefits of each surgical option as well as expected functional outcomes and possible complications.

## Conclusions

Using the largest modern patient cohort to date, this study suggests that patients with high grade osteosarcoma undergoing amputation have significant adverse demographic and clinicopathological features. These include older age, lower income, poorer access to health insurance, larger tumors, and advanced stage. There is an association between limb salvage surgery and improved survival, even when controlling for confounding variables and propensity to receive certain treatments. It is important to note, though, that in database study such as this, it is difficult to infer the causative factors behind such a survival difference. This study also confirms the importance of completing chemotherapy and obtaining negative surgical margins in the treatment of osteosarcoma. Further investigation is necessary to help inform proper decision- making so surgeons can ensure appropriate utilization of limb salvage surgery.

## Data Availability

The data and materials supporting our findings is maintained by the American College of Surgeon through the National Cancer Database; the data is stored securely and access must be requested through an online portal. However this data is publicly accessible by any group affiliated with a Fellow of the American College of Surgeons applying and wishing to gain access. More information may be found at: https://www.facs.org/quality-programs/cancer/ncdb

## Abbreviations

NCDB: National Cancer Database
SEER: National Cancer Institute’s Surveillance, Epidemiology and End Results
OS: Overall Survival
AJCC: American Joint Commission on Cancer
